# Dynamic Nomogram for Predicting Macrovascular Invasion of Patients with Unresectable Hepatocellular Carcinoma after Transarterial Chemoembolization

**DOI:** 10.7150/jca.69548

**Published:** 2022-03-28

**Authors:** Huiwen Yan, Xinhui Wang, Dongdong Zhou, Peng Wang, Zhiyun Yang

**Affiliations:** Center of Integrative Medicine, Beijing Ditan Hospital, Capital Medical University, Beijing 100015, China.

**Keywords:** Hepatocellular carcinoma, Macrovascular invasion, Transarterial chemoembolization, Dynamic Nomogram

## Abstract

**Background:** The purpose of our dynamic nomogram is to help clinical select hepatocellular carcinoma (HCC) patients with transarterial chemoembolization (TACE) treatment advantages.

**Methods:** In total, 1,135 patients with HCC admitted to the Beijing Ditan Hospital of Capital Medical University were enrolled in this study. We used a 7:3 random splits between a training set (n=796) and a validation set (n=339). The dynamic nomogram was established by multiple logistic regression and evaluated by the C-indices. We generated calibration plots, decision analysis curve and a clinical impact curve to assess the clinical usefulness of the nomogram. Macrovascular invasion (MVI) incidence curves were constructed using the Kaplan-Meier method and compared by the log-rank test.

**Results:** Multivariate logistic regression analysis identified six risk factors independently associated with MVI: BCLC staging B vs 0-A (hazard ratio (HR): 2.350, 95% confidence interval (CI): 1.222-4.531; P = 0.010) and staging C vs 0-A (HR: 3.652, 95% CI: 1.212-11.184; P = 0.022), treatment -TACE (HR: 2.693, 95%CI: 1.824-3.987; P < 0.001), tumour size ≥3cm (HR: 2.239, 95%CI: 1.452-3.459; P < 0.001), ɣ-GGT ≥60 (HR: 1.685, 95%CI: 1.100-2.579; P = 0.016), AFP ≥400 (HR: 2.681, 95%CI: 1.692-4.248; P < 0.001) and CRP ≥5 (HR: 3.560, 95%CI: 2.361-5.388; P < 0.001). The C-indices was 0.817 and 0.829 in the training and validation sets, respectively. The calibration curves showed good agreement between the predicted probability and the actual probability by the dynamic nomogram.

**Conclusions:** Our study developed and validated a dynamic nomogram including BCLC staging, treatment modality, tumour size, and three laboratory parameters (ɣ-GGT, AFP and CRP). It has good discrimination and accuracy, and provides a simple and reliable basis for clinical decision-making.

## Introduction

Hepatocellular carcinoma (HCC) is one of the most common malignancies and the third leading cause of cancer-related death 1. A large number of patients do not meet the best indications of hepatectomy and liver transplantation at the time of diagnosis, because HCC is dormant and asymptomatic 2. Transarterial chemoembolization (TACE) is the first choice for patients with unresectable HCC 3. Macrovascular invasion (MVI), including portal vein, hepatic vein and inferior vena cava, is a marker of advanced HCC 4. Studies show that the median overall survival (OS) of HCC patients with portal vein tumour thrombus (PVTT) is 2.7-4 months 5.

Some studies have shown that the increased expression of vascular endothelial growth factor (VEGF) and platelet-derived growth factor receptor (PDGFR) after TACE treatment increases the risk of MVI 6-8. If PVTT occurs after operation, tumour thrombus obstruction will reduce the blood supply of portal vein, affect the dual blood supply of normal liver tissue, and aggravate the liver injury after TACE. Therefore, it is very important to predict the occurrence probability of MVI and explore a better prediction model before TACE. At present, we can only diagnose MVI by ultrasound, computed tomography (CT), magnetic resonance imaging (MRI) and angiography. Nomogram is a practical tool for predicting the occurrence and prognosis of diseases. Compared with other predictive statistical methods, nomogram analysis can provide better individualized prediction risk assessment, and has been widely used in the clinical application of a variety of diseases. At present, most traditional nomograms are still static, need manual calculation, have low repeatability and intelligence. The network-based calculator provides greater convenience, so it is necessary to build a dynamic nomogram model which can predict accurately and is easy to operate.

This study retrospectively studied the clinical characteristics of HCC patients and developed a dynamic nomogram prediction model by logistic regression analysis of independent predictors of MVI. The aim is to predict the individual risk of MVI in HCC patients and to screen patients with the advantage of TACE treatment.

## Material and methods

### Diagnosis and staging of hepatocellular carcinoma

This study retrospectively included 1,135 patients from Capital Medical University Affiliated Beijing Ditan Hospital from January 2008 to December 2018. This study was approved by the Ethics Committee of Beijing Ditan Hospital. According to the principle of randomization, the patients were divided into 7:3, including 796 patients of training set, validation set of 339 patients. HCC is diagnosed by non-invasive criteria used by the European Association for the Study of Liver Diseases (EASL) and the American Association for the Study of Liver Diseases (AASLD)^9^, ^10^. Contrast-enhanced imaging is used to diagnose MVI when the portal vein, hepatic vein, or inferior vena cava show embolic defects and embolic enhancement is the same or similar to primary liver cancer 11. Inclusion criteria were as follows: (1) age ≥18 years; (2) HCC patients diagnosed by imaging or histology evaluation according to the APSAL guidelines 12; (3) receiving TACE. Exclusion criteria were as follows: (1) baseline diagnosis with MVI; (2) patients with metastatic HCC; (3) patients with other tumours; (4) prior HCC-related treatment; (5) patients who were lost to follow-up.

### Demographics and clinical data

Baseline data included patients background such as age, sex, alcohol intake, etiology, and cirrhosis; laboratory data such as white blood cell count (WBC), platelet (PLT), aspartate aminotransferase (AST), alanine aminotransferase (ALT), ɣ-glutamyl transpeptidase (ɣ-GGT) and prothrombin activity (PTA); tumour-related indicators such as alpha-fetoprotein (AFP) and Barcelona Clinic Liver Cancer (BCLC) staging. The observation time of the patient is defined as the time from the first time the patient is included in the study to the occurrence of MVI or the end of follow-up.

### Statistical Analysis

Measurement data were analyzed by t test (normal distribution) and Mann-Whitney U test (non-normal distribution). Categorical variables are shown as numbers and percentages for comparison using the χ^2^ test. Univariate and multivariate logistic regression were used to clarify the independent factors affecting MVI for each variable in the training set. The R was used to draw a nomogram according to the results of multiple factors. The receiver operating characteristic (ROC) curve and the area under the curve (AUC) were used to obtain C-statistics, and the prediction model was evaluated in the training set and the validation set respectively, the degree of calibration of the predictive model was evaluated in both. The clinical impact of the model was evaluated by using the decision curve analysis (DCA) and clinical impact curve (CIC). MVI incidence curves were constructed using the Kaplan-Meier method and compared by log-rank test. P value was less than 0.05. All statistical analyses were performed using R version 4.0.2.

## Results

### Baseline Characteristics

A total of 1,135 patients with HCC diagnosed and treated with TACE were included in our study. The median age of patients in the training set was 57 years old, and the number of male patients was 77.5%. Similar results were obtained in the validation set. Approximately 84.6% of patients overall were infected with hepatitis B Virus (HBV) and 1029 (90.6%) had cirrhosis (Table 1). There was no statistically significant difference in the distribution of variables between the training and validation sets.

### Prognostic factors for MVI

The results of the univariate logistic analysis show that Child-Pugh class (P = 0.004), BCLC staging (P < 0.001), treatment (P < 0.001), tumour number (P < 0.001), tumour size and others obtained from the training sets, were predictors of MVI (Table 2). These factors were included in the multivariate logistic regression analysis (Table 3). Of these, BCLC staging B vs 0-A (hazard ratio(HR): 2.350, 95% confidence interval (CI):1.222-4.531; P = 0.010) and staging C vs 0-A (HR: 3.652, 95%CI: 1.212-11.184; P = 0.022), treatment -TACE ( HR: 2.693, 95%CI: 1.824-3.987; P < 0.001), tumour size ≥3cm (HR: 2.239, 95%CI: 1.452-3.459; P < 0.001), ɣ-GGT ≥60 (HR: 1.685, 95%CI: 1.100-2.579; P = 0.016), AFP ≥400 ( HR: 2.681, 95%CI: 1.692-4.248; P < 0.001) and CRP ≥5 (HR: 3.560, 95%CI: 2.361-5.388; P < 0.001) are independently correlated with MVI.

### Development and validation of an MVI predicting dynamic nomogram

**Figure 1:**
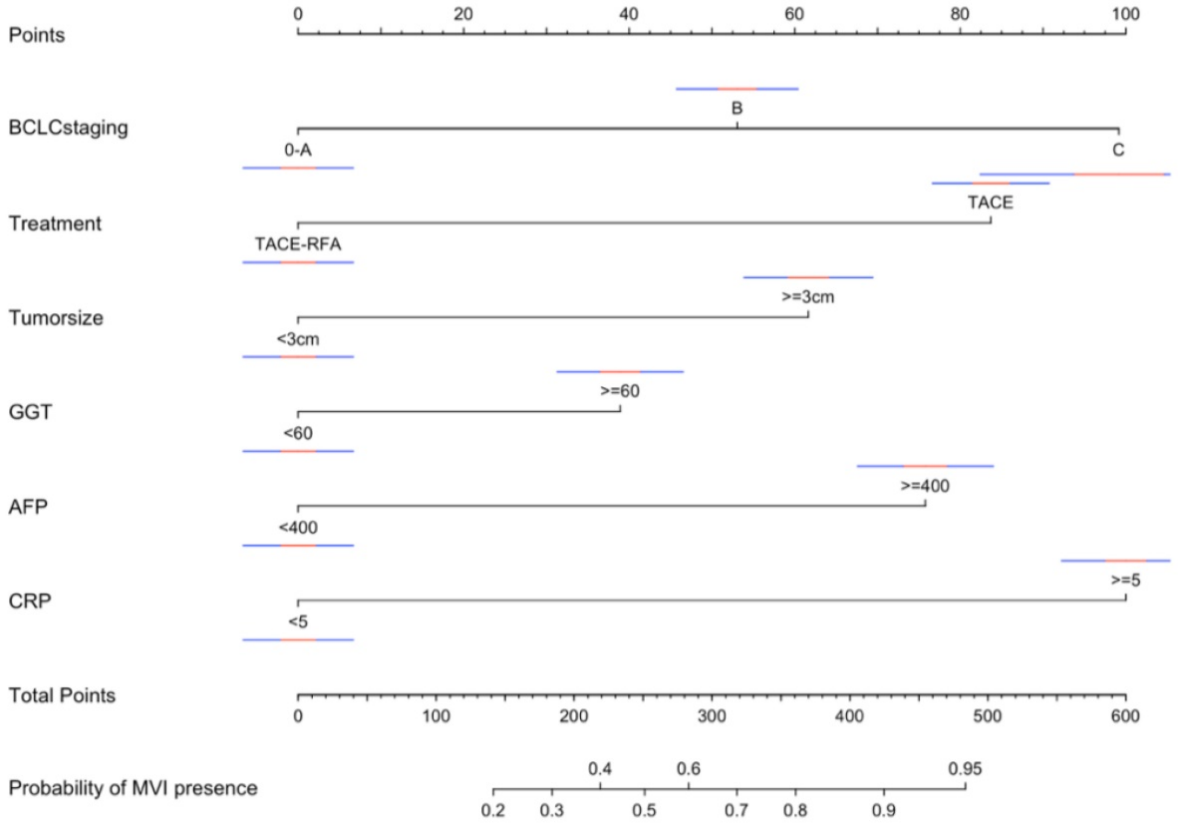
Nomogram for predicting the presence of MVI preoperatively in patients with hepatocellular carcinoma. When using the nomogram, find the position of each variable on the axis and the corresponding point vertically. Then, add the points of all variables, and determine the prediction probability of MVI on the bottom axis. The red and blue line are the confidence interval. MVI, Macroscopic vascular invasion; OR, odds ratio; CI, confidence interval; BCLC, Barcelona Clinic for Liver Cancer; TACE, transcatheter arterial chemoembolization; RFA, radiofrequency ablation; GGT, γ-glutamyl transferase; AFP, α-fetoprotein; CRP, C reactive protein.

**Figure 2:**
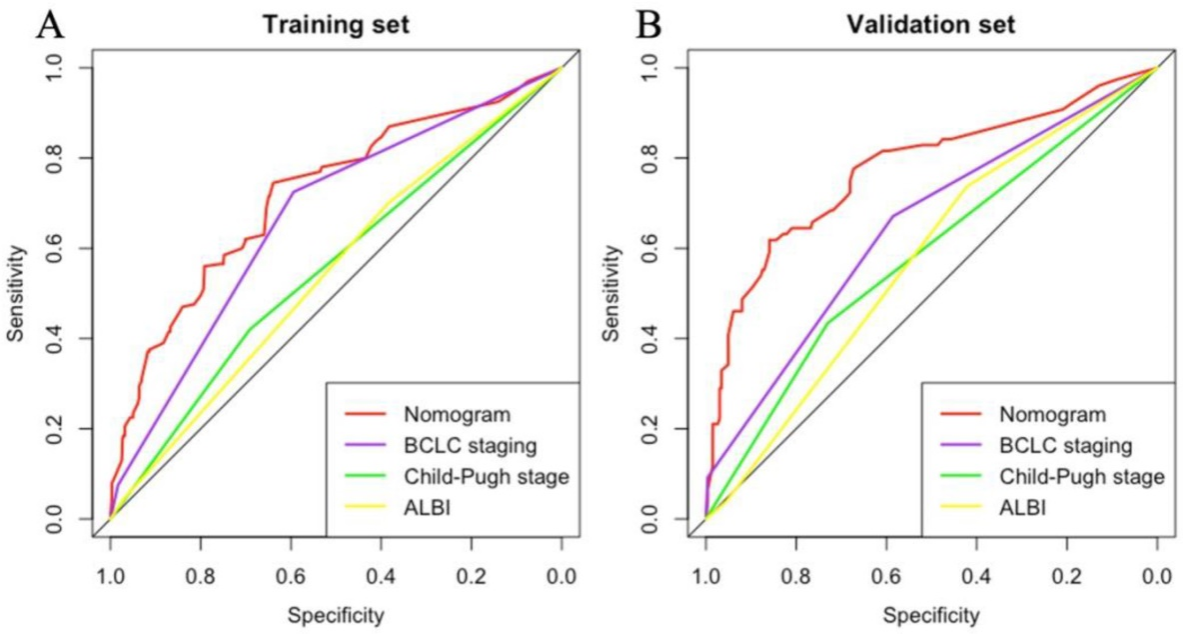
ROC analysis of MVI incidence for the (A) Training set and (B) Validation set. The area under the curve (AUC) of the scoring model developed in the study was greater than that of a single indicator. ROC, receiver operating characteristic; AUC, area under the curve.

**Figure 3:**
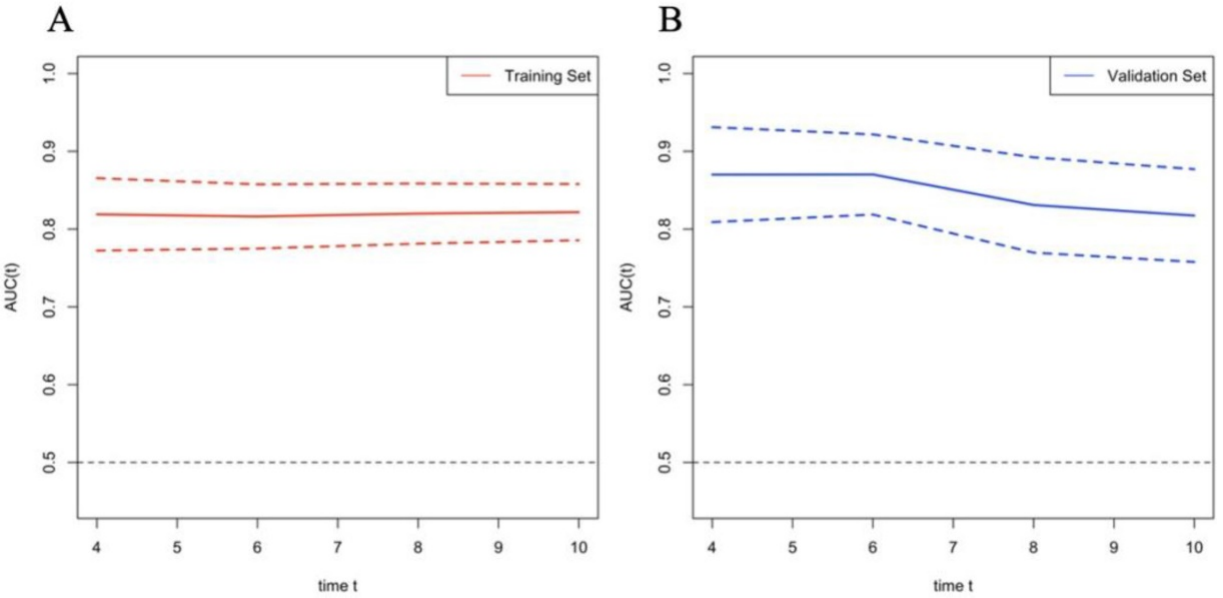
Time-dependent AUROC values of the current mode. (A) Time-dependent AUROC values in Training set; (B) Time-dependent AUROC values in Validation set. AUROC, area under receiver operating characteristic curve.

According to the variables of multiple factors, the indicators included in nomogram are BCLC staging, treatment, tumour size, ɣ-GGT, AFP and CRP (Figure 1). When using the nomogram, find the position of each variable on the axis and correspond to the points scale above the nomogram in the vertical direction, so as to obtain the single score of this factor, and then add the scores of all variables to obtain the total points of each patient. According to the total points of each patient, it corresponds to the probability of MVI presence. In other words, the higher the total point, the higher the probability of MVI. In addition, to facilitate the use of dynamic nomogram by clinicians, a web-based interface (https://hccnomogran.shinyapps.io/MVINomogram/) was created using the "Dynnom" software package to calculate the probability of MVI in HCC patients (Figure S1). Nomogram showed good accuracy in estimating MVI risk, with a C-index of 0.817 in the training set and 0.829 in the validation set. The specificity, sensitivity, positive and negative predictive values, positive and negative likelihood ratio were 78.5%, 74%, 53.6%, 90.0%, 3.4 and 0.3 in the training set, 78.7%, 76.3%, 46.5%, 89.8%, 3 and 0.4 in the validation set (Table 4). In the ROC curve analysis, compared with other classical models, the AUC of this model is larger. It shows that the nomogram model has good discrimination (Figure 2A, B). The current model has significantly higher 1-year AUC value (Figure 3A, B), indicating good model performance. The Classification and Regression Tree (CART) in Figure S2A-B also reflects the validity of the model indicators.

### Clinical usefulness of risk prediction dynamic nomogram

**Figure 4:**
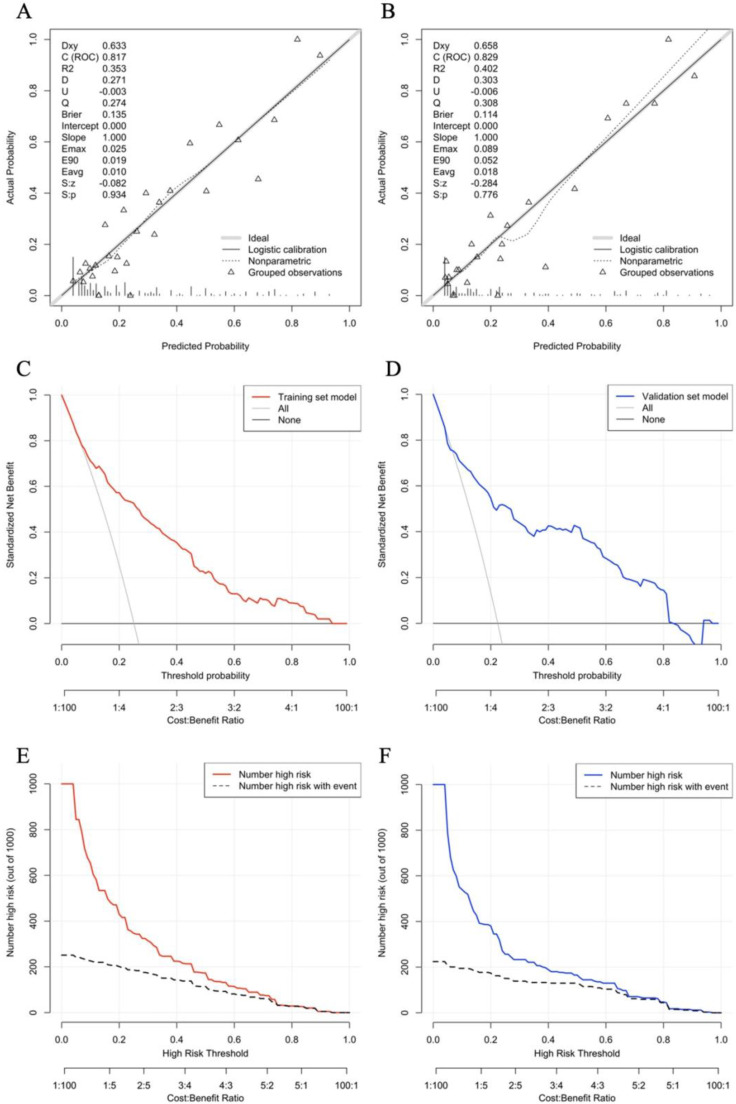
Evaluate the prediction effect of nomogram in the Training (A,C,E) and Validation (B,D,F) set. (A,B) Calibration plot, (C,D) decision curve and (E,F) clinical impact curve of the nomogram for critical probability in the HCC patients, in which the predicted critical probability was compared well with the actual probability and had superior standardized net benefit.

Further analysis shows that the nomogram prediction model has excellent discrimination evaluation performance. The calibration curve reflects the degree of agreement between the model predictions and the actual values. By plotting the calibrations, we readily found good agreement between the frequencies observed in the two datasets and the predicted probabilities of MVI patients (Fig. 4A, B). Decision Curve Analysis (DCA) was used to assess the availability and effectiveness of the predictive model, using this nomogram to predict MVI may bring more benefits than treatment of all patients or no treatment of any patient (Fig. 4C, D). We used a red curve and a blue curve (high risk number) to represent the number of people in both sets classified as positive (high risk) at each threshold probability; the gray dashed line is the true positive number for each threshold probability. The CIC suggests that individualization with the model has an important impact on clinical benefit (Fig. 4E, F).

### Stratifying patients according to risk

**Figure 5:**
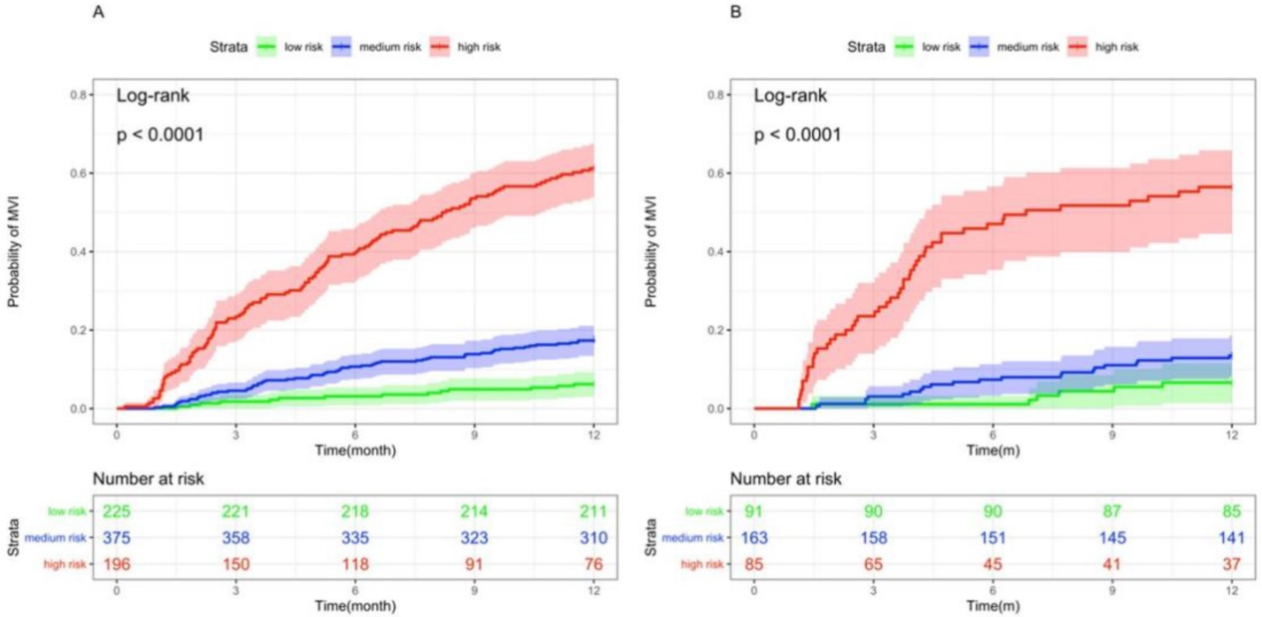
Kaplan-Meier curves of risk group stratification for MVI occurrence in the (A)Training set and (B)Validation set.

Based on the dynamic nomogram we developed in this study, we divided patients into low, medium and high risk groups, and the incidence of MVI was significantly lower in the medium and low risk groups than in the high risk group, both in the training and validation sets (p < 0.001, Figure 5).

## Discussion

Vascular invasion can be divided into microvascular invasion and macrovascular invasion. Macrovascular invasion is the result of the gradual development of microvascular invasion. Previous studies 13-16 mainly focused on the incidence of microvascular invasion, because microvascular invasion is a marker of postoperative recurrence, while macrovascular invasion focuses on the prognosis and survival of patients with HCC. Current guidelines show that first-line treatment is not enough to prevent macrovascular invasion, and the BCLC staging treatment model only recommends TACE for some patients with BCLC stage B. However, the BRIDGE Institute reported that the study included 18,031 patients (67% in Asia) and that TACE was the most used treatment for patients with BCLC C staging 17. Obviously, in the East, TACE is considered to provide acceptable tolerance and favorable survival benefits for patients with unresectable HCC. Therefore, our study explored a better dynamic nomogram model for these patients without vascular invasion to predict the risk of MVI.

Recently, a retrospective propensity-matched study comparing TACE- radiofrequency ablation (RFA) and TACE alone for advanced HCC with macrovascular disease demonstrated that TACE-RFA had better survival than TACE alone in patients with HCC 18. This supports our clinical finding that treatment modality is an important factor associated with MVI risk. In this study, patients who received TACE+RFA (534 of 796 [67.1%] and 207 of 339 [61.1%], respectively) significantly prolonged the occurrence of MVI in HCC. When combined with RFA, TACE can detect microsatellite foci, mark the extent and size of the tumour and contribute to subsequent RFA Treatment. Prior TACE embolisation chemotherapy can not only significantly reduce the extent of the tumour lesion and provide a more precise localisation of RFA, but also block the blood supply to the lesion, effectively reduce the effect of the heat sink effect, improve the ablation treatment effect, and avoid the loss of embolic drugs during TACE treatment, thereby enhancing the sustained therapeutic effect of TACE 19.

In this study, AFP ≥400 ng/ml was found to be a risk factor for MVI, and elevated AFP levels were associated with MVI. Schlichtemeier et al. investigated 125 patients with HCC and showed that serum AFP ≥400 ng/ml was an independent risk factor for MVI 20. It has been shown that the preoperative peripheral blood count of circulating tumour cells in patients with HCC is strongly associated with MVI 21. Positive AFP mRNA expression in circulating tumour cells is a key predictor of vascular infiltration and metastasis in HCC, explaining the relationship between elevated AFP and MVI 22. One study suggested that AFP promotes the expression of PDL1 and causes immune escape of hepatoma cells 23.

In this study, ɣ-GGT >60U/L was strongly associated with MVI, a ubiquitous epithelial enzyme whose levels increase in response to the presence of free radicals as ɣ-GGT is involved in redox regulation, which in turn is thought to be associated with tumour growth^24^, ^25^. Elevated ɣ-GGT levels are associated with vascular invasion, tumour size, number of tumours and AFP levels 26-28. For solitary HCCs of ≤ 5 cm, ɣ-GGT > 53U/L is strongly associated with the development of MVI 29.

CRP, which is synthesised by interleukin 6, is mainly used as a marker of inflammation 30. In advanced HCC, CRP affects the overall survival of patients after TACE 31. Increasingly, it is recognised that inflammatory markers such as CRP are significantly correlated with the aggressiveness of HCC and are useful predictors of vascular invasion 28, 32.

Our study has some limitations. First, all data analysed in this study were from a single institution, and although the sample size of this study was large, data from other centres are still needed to further validate the model. Secondly, all TACE and RFA procedures were performed at a single institution. Therefore, the experience of physicians may influence the results of the study. Thirdly, unlike studies in the USA, Japan and Europe, HCC in China is mainly related to HBV and has different tumour characteristics; therefore, it is necessary to further explore treatment strategies for patients with HCC in the above-mentioned areas.

In conclusion, our study developed and validated a dynamic nomogram including BCLC staging, treatment modality, tumour size, and three laboratory parameters (ɣ-GGT, AFP and CRP), to predict the occurrence of MVI in patients with unresectable HCC. It has good discrimination and accuracy, and provides a simple and reliable basis for clinical decision-making.

## Supplementary Material

Supplementary figures.

## Figures and Tables

**Table 1:** Clinical characteristics of the HCC patients.

Characters		Training set	Validation set	*P*
		n=796 (%)	n=339 (%)	value
**Patients background**				
Age(years)		56.98 ± 10.08	56.68 ± 9.77	0.645
Sex	Female	179 (22.5)	76 (22.4)	1
	Male	617 (77.5)	263 (77.6)	
History of alcohol use	No	505 (63.4)	210 (61.9)	0.682
	Yes	291 (36.6)	129 (38.1)	
Family history of HCC	No	768 (96.5)	322 (95.0)	0.309
	Yes	28 (3.5)	17 (5.0)	
Etiology	Others	113 (14.2)	62 (18.3)	0.097
	HBV	683 (85.8)	277 (81.7)	
Treatment	TACE-RFA	534 (67.1)	207 (61.1)	0.06
	TACE	262 (32.9)	132 (38.9)	
Cirrhosis	No	74 (9.3)	32 (9.4)	1
	Yes	722 (90.7)	307 (90.6)	
Child-Pugh stage	A	528 (66.3)	235 (69.3)	0.361
	B	268 (33.7)	104 (30.7)	
**Laboratory data**				
WBC(10^9/L)	<4	358 (45.0)	135 (39.8)	0.124
	≥4	438 (55.0)	204 (60.2)	
Neutrophils(10^9/L)		2.42 (1.67, 3.58)	2.60 (1.75, 3.69)	0.133
Lymphocytes(10^9/L)		1.10 (0.76, 1.60)	1.16 (0.78, 1.71)	0.077
NLR	<1.86	465 (58.4)	198 (58.4)	1
	≥1.86	331 (41.6)	141 (41.6)	
RBC(10^12/L)	<4	353 (44.3)	142 (41.9)	0.484
	≥4	443 (55.7)	197 (58.1)	
HGB(g/L)	<120	226 (28.4)	104 (30.7)	0.481
	≥120	570 (71.6)	235 (69.3)	
PLT(10^9/L)	<100	428 (53.8)	170 (50.1)	0.292
	≥100	368 (46.2)	169 (49.9)	
ALT(U/L)	<50	609 (76.5)	250 (73.7)	0.359
	≥50	187 (23.5)	89 (26.3)	
AST(U/L)	<40	469 (58.9)	203 (59.9)	0.813
	≥40	327 (41.1)	136 (40.1)	
TBIL(umol/L)	<18.8	501 (62.9)	208 (61.4)	0.662
	≥18.8	295 (37.1)	131 (38.6)	
ALB(g/L)	<40	536 (67.3)	218 (64.3)	0.357
	≥40	260 (32.7)	121 (35.7)	
ɣ-GGT(U/L)	<60	480 (60.3)	209 (61.7)	0.719
	≥60	316 (39.7)	130 (38.3)	
Cr(mg/dL)	<111	777 (97.6)	332 (97.9)	0.908
	≥111	19 (2.4)	7 (2.1)	
PTA(%)	<70	189 (23.7)	79 (23.3)	0.934
	≥70	607 (76.3)	260 (76.7)	
CRP(mg/L)	≤5	578 (72.6)	251 (74.0)	0.672
	>5	218 (27.4)	88 (26.0)	
**Tumour-related indicators**				
AFP(ng/mL)	<400	653 (82.0)	269 (79.4)	0.329
	≥400	143 (18.0)	70 (20.6)	
Tumour number	Solitary	495 (62.2)	222 (65.5)	0.323
	Multiple	301 (37.8)	117 (34.5)	
Tumour size(cm)	≤3	458 (57.5)	207 (61.1)	0.3
	>3	338 (42.5)	132 (38.9)	
BCLC staging	0-A	409 (51.4)	179 (52.8)	0.736
	B	362 (45.5)	152 (44.8)	
	C	25 (3.1)	8 (2.4)	

**Table 2:** Univariate Logistic Regression Analysis of MVI Presence Based on Preoperative Data in the Training set

Variable	OR	95%CI		P value
Age(years)	0.992	0.977	1.008	0.345
Sex, Male	1.038	0.710	1.539	0.849
Alcohol use, Yes	1.287	0.925	1.785	0.132
Family history of HCC, Yes	1.200	0.490	2.673	0.669
Etiology, HBV	1.022	0.652	1.643	0.927
Child-Pugh class, B	1.621	1.164	2.254	0.004
Treatment, TACE	3.675	2.635	5.146	<0.001
Cirrhosis, Yes	1.819	0.993	3.612	0.067
WBC(10^9/L), ≥4	1.175	0.851	1.627	0.329
Neutrophils(10^9/L)	1.108	1.018	1.206	0.017
Lymphocytes(10^9/L)	0.765	0.582	0.993	0.049
NLR, ≥1.86	1.810	1.310	2.502	<0.001
RBC(10^9/L), ≥4	0.758	0.549	1.045	0.091
HGB(g/L), ≥120	0.532	0.379	0.749	<0.001
PLT(10^9/L), ≥100	0.961	0.696	1.325	0.282
ALT(U/L), ≥50	1.243	0.856	1.789	0.247
AST(U/L), ≥40	1.966	1.423	2.722	<0.001
TBIL(umol/L), ≥18.8	1.215	0.873	1.685	0.245
ALB(g/L), ≥40	0.723	0.505	1.025	0.073
ɣ-GGT (U/L), ≥60	2.893	2.085	4.031	<0.001
Cr(mg/dL), ≥111	2.216	0.847	5.554	0.092
PTA(%), ≥70	0.603	0.422	0.865	0.006
CRP(mg/L), >5	5.523	3.908	7.842	<0.001
AFP(ng/ml), ≥400	3.080	2.104	4.503	<0.001
Tumour number, Multiple	2.020	1.460	2.799	<0.001
Tumour size(cm), ≥3	4.145	2.956	5.864	<0.001
BCLC staging				
B vs 0-A	3.607	2.539	5.180	<0.001
C vs 0-A	9.655	4.177	23.246	<0.001

**Table 3:** Multivariate Logistic Regression Analysis of MVI Presence Based on Preoperative Data in the Training set

Variable	OR	95%CI		P Value
BCLC staging				
B vs 0-A	2.350	1.222	4.531	0.010
C vs 0-A	3.652	1.212	11.184	0.022
Treatment, TACE	2.693	1.824	3.987	<0.001
Tumour size, ≥3cm	2.239	1.452	3.459	<0.001
γ-GGT, ≥60	1.685	1.100	2.579	0.016
AFP, ≥400	2.681	1.692	4.248	<0.001
CRP, ≥5	3.560	2.361	5.388	<0.001

**Table 4:** Accuracy of the Prediction Score of the Nomogram for Estimating the Risk of MVI Presence

Variable	Training set	Validation set
Area under ROC curve, concordance index	0.817	0.829
Sensitivity, %	74	76.3
Specificity, %	78.5	78.7
Positive predictive value, %	53.6	46.5
Negative predictive value, %	90.0	89.8
Positive likelihood ratio	3.4	3
Negative likelihood ratio	0.3	0.4
