# Supplementary material for: Dynamic Nomogram for Predicting Macrovascular Invasion of Patients with Unresectable Hepatocellular Carcinoma after Transarterial Chemoembolization
Source: J Cancer. 2022 Mar 28;13(6):1914–22. doi: 10.7150/jca.69548 (PMC8990408; doi:10.7150/jca.69548)
Supplement: Supplementary file 1 — Supplementary figures. [file jcav13p1914s1.pdf]

Figure S1.

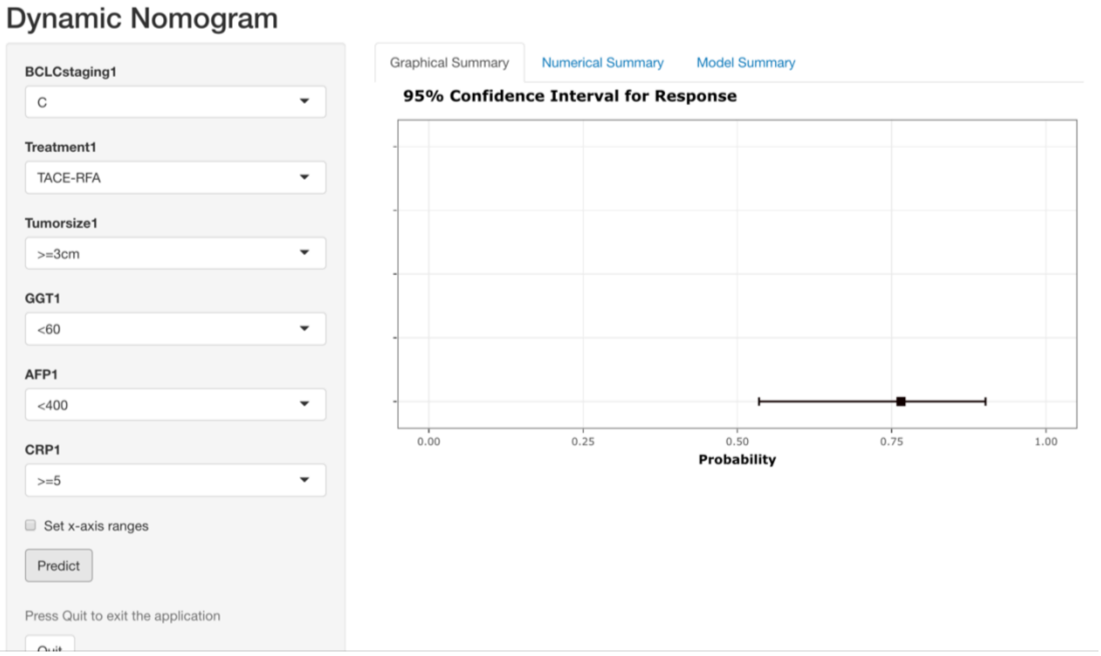

Fig. S1. The dynamic Nomogram schematic.

Figure S2.

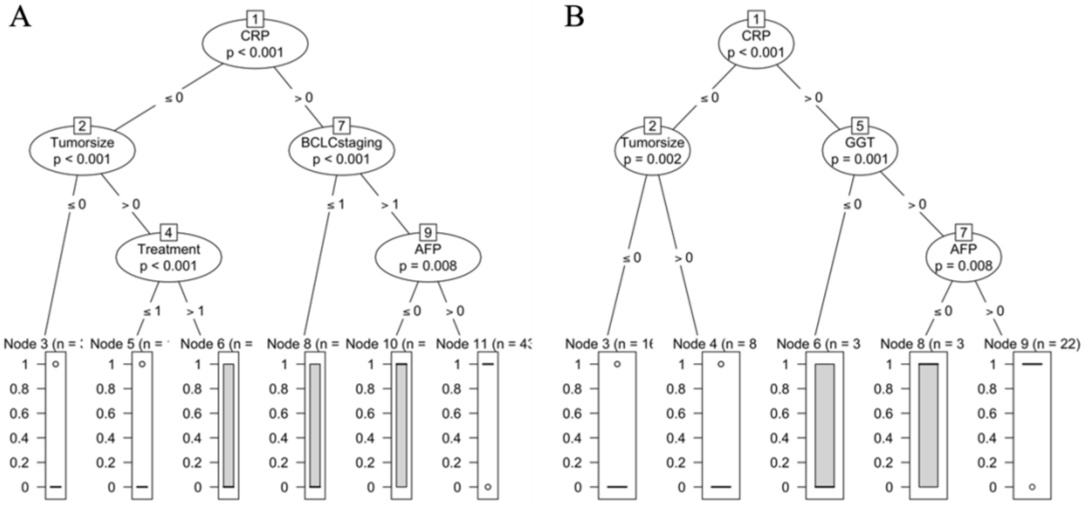

Fig. S2. The Classification and Regression Tree(CART) for predicting the presence of MVI preoperatively in patients with hepatocellular carcinoma. (A)Training set; (B)Validation set.
